# 1432. Touch-Free Automatic Alcohol-Based Hand Rub Dispensers in Healthcare Settings: A Simulation Study of Dispenser Energy Impact on Hand Hygiene Disruption

**DOI:** 10.1093/ofid/ofad500.1269

**Published:** 2023-11-27

**Authors:** Nanshan Chen, James W Arbogast, John J McNulty, Paul J Brown, Demetrius Henry, Susan O’Hara, Abedallah Al Kader, Angela Hu, Theodore T Allen, Cathy H Xia

**Affiliations:** The Ohio State University, Columbus, Ohio; GOJO Industries, Inc., Akron, Ohio; GOJO Industries, Inc., Akron, Ohio; GOJO Industries, Inc., Akron, Ohio; GOJO Industries, Inc., Akron, Ohio; The Ohio State University, Columbus, Ohio; The Ohio State University, Columbus, Ohio; Columbus Academy, Gahanna, Ohio; The Ohio State University, Columbus, Ohio; The Ohio State University, Columbus, Ohio

## Abstract

**Background:**

Hand hygiene (HH) matters because it decreases pathogen transmission that can cause infection. Automatic alcohol-based hand rub (ABHR) dispensers are widely adopted in healthcare facilities as the preferred means of HH. Traditional automatic dispensers have a large supply of batteries in the dispenser housing, whereas energy-on-the-refill (EOR) is a newer power supply solution, consisting of a relatively small battery attached to a refill bottle. The objective of this study was to assess dispenser design impact on missed HH opportunities and facility workflow disruption by mitigating battery maintenance.

**Methods:**

We used date-driven discrete event simulation to evaluate the performance of three leading types of automatic dispensers in four common types of hospitals (Table 1). We analyzed up to 8 years of historical usage data and identified the usage pattern, which are used as the input traffic for our simulation model. Dispenser energy performance parameters were inputs to measure the workflow disruption of the different types of dispensers over a 6-year period in terms of battery replacements, duration of downtime, and the number of missed HH opportunities.

**Table 1:**
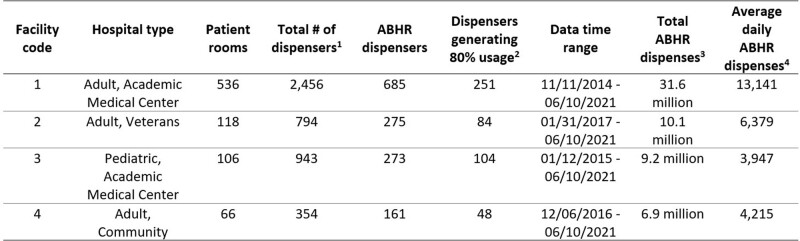
Summary of facility information and dispense event details to inform modeling.

1. This total included soap dispensers used for hand washing and ABHR dispensers that were not equipped with automated monitoring (e.g., those provided in office areas without patients). 2. These were the dispensers used in the analysis and modeling results. 3. Total dispenses for all ABHR dispensers during the entire time range. 4. This is the average number of ABHR dispenses daily for the facility for all dispensers.

**Results:**

The simulation results suggested that dispensers with EOR technology were free of battery failures over the entire 6 years, and thus 0 HH misses were incurred due to dead batteries (Figure 1). All other designs had a significant amount HH misses due to battery failures, ranging from 2,514 (± 547) to 40,522 (± 4,506) per facility. However, the majority of HH misses were caused by empty ABHR refills. The maximum number of battery change events was 802 (± 0.60).

**Figure 1:**
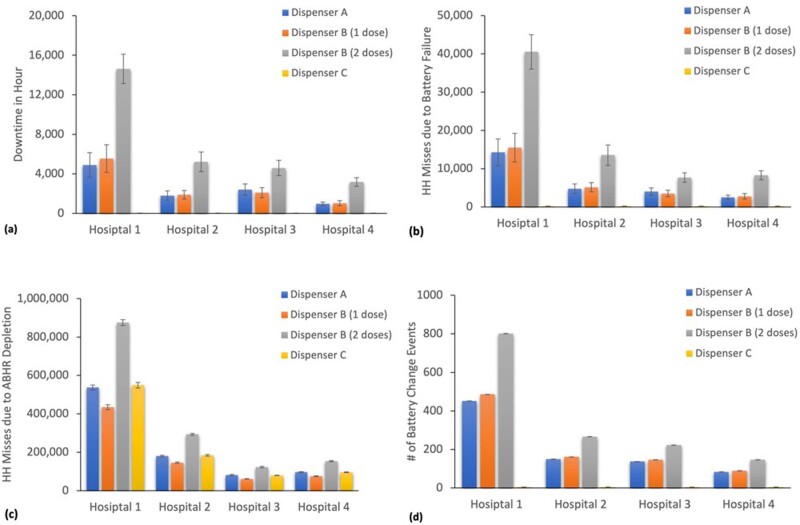
Modeling results for each hospital and all dispenser types1 over the 6-year period simulation for the ABHR dispensers that comprise 80% of usage based on prior data. This figure displays (a) total dispenser downtime in hours, (b) the total number of HH misses due to battery failures, (c) the total number of HH misses due to ABHR availability (assuming 12 hours between an empty refill being replaced), and (d) the total number of battery change-out events. 95% CIs were also illustrated but might be unnoticeable, as they are on a much smaller scale compared to the mean.

1. Dispenser A is a traditional design with 4 “D” cell batteries in the housing and has an average dispense energy of 2.02 J/ml. Dispenser B is a traditional design with 3 “D” cell batteries in the housing and has an average dispense energy of 1.78 J/ml. Dispenser C is a new design with a “AA” battery on the refill and has an average dispense energy of 1.78 J/ml. Dispenser B is modeled at 1 dose and 2 doses because it has some ABHR formulations that can require 2 dispenses to meet the Healthcare Personnel Handwash test method antimicrobial efficacy success criteria.

**Conclusion:**

Differences in dispenser design, including the energy management system and usage profiles have significant impact on HH performance, which in turn can affect infection risk. By adopting the EOR system, facilities can effectively eliminate the need for battery maintenance, resulting in labor and workflow efficiencies. The EOR system significantly reduces HH disruptions and may decrease complaints by caregivers, patients and visitors. Importantly facilities should carefully study dispenser usage patterns to implement optimized policies and practices for placement and refill maintenance of ABHR dispensers to minimize overall missed HH opportunities.

**Disclosures:**

**Nanshan Chen, n/a**, GOJO Industries, Inc.: Grant/Research Support **James W. Arbogast, PhD**, GOJO Industries, Inc.: employee **John J. McNulty, n/a**, GOJO Industries, Inc.: employee **Paul J. Brown, n/a**, GOJO Industries, Inc.: employee **Demetrius Henry, n/a**, GOJO Industries, Inc.: employee **Susan O'Hara, PhD**, GOJO Industries, Inc.: Grant/Research Support **Abedallah Al Kader, n/a**, GOJO Industries, Inc.: Grant/Research Support **Angela Hu, n/a**, GOJO Industries, Inc.: Grant/Research Support **Theodore T. Allen, PhD**, GOJO Industries, Inc.: Grant/Research Support **Cathy H. Xia, PhD**, GOJO Industries, Inc.: Grant/Research Support

